# Heroin-Induced Transverse Myelitis in a Chronic Heroin User: A Case Report

**DOI:** 10.7759/cureus.41286

**Published:** 2023-07-02

**Authors:** Mueez Hussain, Drake Shafer, Joseph Taylor, Ranjit Sivanandham, Hellen Vasquez

**Affiliations:** 1 Internal Medicine, Southwest Healthcare MEC, Temecula, USA; 2 Internal Medicine, California University of Science and Medicine, Colton, USA

**Keywords:** central nervous system, case report, substance use, neuroimaging, paralysis, heroin, transverse myelitis

## Abstract

Transverse myelitis is a rare but documented sequela of heroin use. While the underlying etiology is not clearly elucidated, the prevailing pathophysiologic mechanism amongst existing literature suggests an immune-mediated hypersensitivity reaction due to heroin insufflation following a long period of abstinence. Outcomes vary among the limited reports, but prognosis tends to be poor due to an acute and rapidly progressive disease course. Here, we describe a case of extensive transverse myelitis in a chronic heroin user following heroin insufflation. This report hopes to provide greater insight into the underlying cause of this rare phenomenon due to our patient’s discrepancy from the documented norm of heroin abstinence preceding disease onset.

## Introduction

Transverse myelitis (TM) is a rare neurological condition characterized by inflammation and damage to the spinal cord with potentially severe long-term neurological morbidity and mortality. The etiology of TM varies, including a large spectrum of underlying causes including, but not limited to, drug/toxin, infectious, paraneoplastic, idiopathic, systemic autoimmune disorders, and acquired demyelinating diseases [1-2]. Quick identification is imperative to improve long-term effects. Documented only in limited case reports, acute TM due to heroin use is a rare phenomenon usually affecting the cervical and thoracic portions of the spinal cord and characterized by rapid symptom onset over the course of hours to days [3-4]. Although there are reports of heroin-induced TM following intravenous use, most cases report TM following heroin insufflation [3-9]. Leading understanding supports an immune-mediated hypersensitivity as the underlying pathophysiology due to previous accounts reporting an inciting event of heroin use following a long period of abstinence [3-9]. Our case presents a unique situation where heroin insufflation-induced TM occurred in a consistent heroin user. Through reporting on this deviation from previous cases, we hope to better advance the understanding of the etiology behind heroin-induced TM.

## Case presentation

A 50-year-old man with a past medical history of heroin use and hypertension presented to the Emergency Department (ED) with acute onset right-sided focal weakness. The patient reported smoking heroin earlier that day and then napping for 4 hours. Shortly after waking, the patient noticed right-sided weakness with difficulty moving the right upper and lower extremities. The severity of the weakness became progressively worse, at which point the patient came to the ED. The symptom onset was roughly 5 hours after heroin insufflation. The patient denied any similar problems in the past and endorsed being a consistent heroin user. Past medical history was significant for consistent heroin use, hypertension, obesity, diabetes mellitus type 2, and hepatitis C. Vital signs were within normal limits. The initial physical exam showed weakness in the right upper and lower extremities with no sensory changes. Re-evaluation two hours after admission showed progressive weakness now spreading to the left extremities. Widespread loss of sensation and rapidly progressing paralysis soon occurred, ultimately requiring intubation due to decreased respiratory effort. 

**Figure 1:**
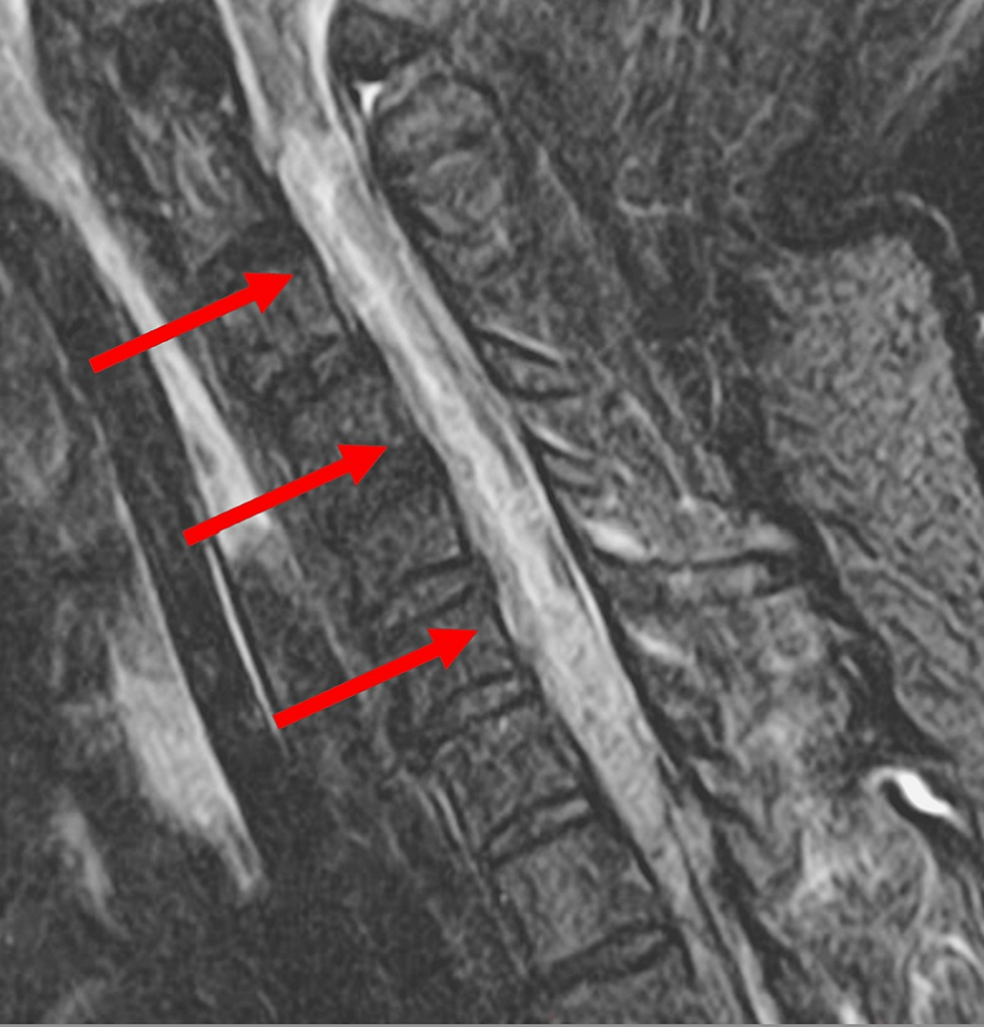
Transverse Myelitis visualized on MRI Red arrows point to abnormal T2 signaling and increased cord expansion extending from C2-C7.

Diagnosis of TM was confirmed with an MRI of the cervical spine revealing abnormal T2 signaling extending multiple levels down the central spinal cord tracts from C2-C7 with increased cord expansion suggestive of transverse myelitis (Figure 1). This was again demonstrated on a repeat MRI C-spine with contrast. An MRI of the brain was performed, indicating no abnormal findings or pathological changes. The rarity of the disease posed a diagnostic challenge as multiple other causes remained on the differential, including but not limited to infection, ischemia, syrinx, paraneoplastic, and autoimmune. An extensive diagnostic work-up was conducted to rule out other potential causes. A lumbar puncture was obtained and returned unremarkable. Plasmapheresis was initiated to empirically manage autoimmune transverse myelitis but was discontinued after 3 days per neurology recommendations as negative anti-NMO (neuromyelitis optica) and anti-MOG (myelin oligodendrocyte glycoprotein) antibodies returned, indicating no evidence of autoimmune transverse myelitis. The serum autoimmune panel was negative.

Neurology was consulted for continued management. Our patient received a 5-day course of high-dose steroids with 1g IV methylprednisone. Unfortunately, his symptoms did not improve following high-dose steroids. During a neurological exam with the patient not on sedation, there was complete paralysis and loss of sensation in all extremities. The patient was able to blink and stick out his tongue in response to commands. Before intubation, the patient had a limited verbal response of grunting. The patient’s course was complicated by persistent rhabdomyolysis with elevated creatine kinase (CK) causing worsening renal failure, ultimately leading to the patient requiring hemodialysis. Eventually, a tracheostomy and percutaneous endoscopic gastrostomy tube were placed, given his continued ventilator dependency and inability to swallow. The patient was eventually discharged to a long-term acute care facility for extensive rehabilitation.

## Discussion

To our knowledge, roughly 10 cases of heroin-induced TM are available in the scientific literature over approximately the last 50 years. Strong similarities have been noted between cases including most being secondary to heroin insufflation, disease onset following use after a period of abstinence, and the presence of rapid onset of symptoms often resulting in permanent neurological deficits and death. While high-dose steroids have been successful in halting progression in some patients, the prognosis is usually poor [3-9]. 

A key difference in drug use history was present in our patient which may shed light on various theories as to why heroin-induced TM occurs. The greatest consistency amongst previous cases is heroin insufflation in a previous user following a period of non-use. This documented timeline strongly suggests an immune-mediated hypersensitivity reaction as being the underlying cause of heroin-induced TM. Ivanosvki et al clearly demonstrated this association by testing for opioid use through hair sampling to support a period of previous abstinence in their patient [8]. Through our patient’s verbal history upon initial presentation and corroboration by close friends, a history of consistent heroin use was evident. Of note, our patient’s MRI findings also displayed severe spinal cord involvement from C2-C7 which is more extensive than most other reported cases likely attributing to the lack of efficacy in high-dose steroid treatment. 

Many different hypotheses have been proposed for the underlying mechanism behind heroin-induced TM, including vascular damage leading to ischemia, compression, direct/contaminant toxicity, and immunological effects from heroin or associated contaminants [3-8]. While our reported case presents an outlier due to consistent heroin use, it is important to note that this does not disprove the leading autoimmune-mediated theory but provides further evidence to help guide our understanding of this rare heroin-associated sequela. 

Ischemia due to poor vascular supply has also been proposed as a possible mechanism [5,7,10]. Consistent with this theory in our patient is the 4-hour nap following heroin use, which may have been accompanied by respiratory depression, hypotension, and a long period of neck hyperextension contributing to poor blood flow. However, inconsistent with the vascular theory, our patient had normal vitals on arrival with C2-7 being primarily affected as it is more understandable for areas with less collateral supply such as the thoracic region to be preferentially affected by poor blood flow [5,7,10-11].

## Conclusions

Our report highlights the difficult diagnostic challenge of heroin-induced transverse myelitis and furthers our understanding of clinical features, diagnostics, treatment, and prognosis. As heroin-induced TM progresses rapidly, fast diagnosis and treatment are imperative. Obtaining a detailed patient history, thorough neurologic exam, and imaging is essential to diagnosis. Although there is limited efficacy, treatment with high-dose steroids is the recommended plan of action. Due to our case’s unique spontaneous onset of transverse myelitis during consistent heroin use, we would like to emphasize the impact this case report may have on the current understanding of the pathophysiology behind heroin-induced transverse myelitis as we compare autoimmune, vascular, and other etiologies. 
